# Airway epithelial TSLP production of TLR2 drives type 2 immunity in allergic airway inflammation

**DOI:** 10.1002/eji.201847663

**Published:** 2018-10-12

**Authors:** Jiajia Lv, Qianying Yu, Jie Lv, Caixia Di, Xiaoliang Lin, Wen Su, Min Wu, Zhenwei Xia

**Affiliations:** ^1^ Department of Pediatrics Ruijin Hospital affiliated to Shanghai Jiao Tong University School of Medicine Shanghai China; ^2^ Department of Pediatrics Xinhua Hospital affiliated to Shanghai Jiao Tong University School of Medicine Shanghai China; ^3^ School of Medicine & Health Sciences Department of Biomedical Sciences University of North Dakota Grand Forks North Dakota

**Keywords:** TSLP, TLR2, signaling pathways, basophils, type 2 immune responses

## Abstract

Epithelial cells (ECs)‐derived cytokines are induced by different stimuli through pattern recognition receptors (PRRs) to mount a type‐2‐cell‐mediated immune response; however, the underlying mechanisms are poorly characterized. Here, we demonstrated asthmatic features in both primary bronchial epithelial cells (pBECs) and mouse model using several allergens including ovalbumin (OVA), house dust mite (HDM), or *Alternaria alternata*. We found that toll‐like receptor 2 (TLR2) was highly induced in ECs but not dendritic cells (DCs) by various allergens, leading to recruitment of circulating basophils into the lung via C‐C chemokine ligand‐2 (CCL2). TLR2 expression increased thymic stromal lymphopoietin (TSLP) production through the NF‐κB and JNK signaling pathways to extend the survival of recruited basophils and resident DCs in the lung, predisposing a type‐2‐cell‐mediated airway inflammation. Conversely, TLR2 deficiency impaired http://secretion of TSLP and CCL2, decreased infiltration of lung basophils, and increased resistance to Th2 response. Blocking TSLP also phenocopied these phenomena. Our findings reveal a pro‐inflammatory role of airway ECs through a TLR2‐dependent TSLP production, which may have implication for treating allergic asthma.

## Introduction

More than two billion people globally suffer from allergic diseases. These inflammatory disorders closely resemble each other in that they cause mucosal barriers to mount a Th2 response involving mast cells, eosinophils, basophils, and DCs with structural and functional changes 1, 2.

In the airway, triggering of PRRs on epithelial cells (ECs) induces the release of chemokines and cytokines to attract and activate immune cells, leading to Th2‐cell‐associated asthma features 3. TLRs act as the first line of mucosal defense to recognize, process, and eliminate invading microorganisms 4. For example, TLR4 recognizes house dust mite (HDM) and the proteolytic activities of the allergen drive type 2 responses via inducing protease‐activated receptor 2 (PAR2) 5, 6. In addition, nucleotide‐binding oligomerization domain (NOD) proteins are important intracellular PRRs that sense the intracellular bacterial products and regulate type 2 immune responses partly by negatively modulating TLR signaling 7.

Sensing of allergens by PRRs on barrier ECs stimulates the production of prototypical cytokines, providing essential signals to elicit type 2 responses by facilitating DCs, basophils, and innate lymphoid cell (ILC) responses 8, 9, 10, 11. Among these cytokines, thymic stromal lymphopoietin (TSLP) appears to play a critical role in the activation of immune cells 12. Expression of TSLP mRNA and protein has been shown to be significantly increased in airway epithelia from asthmatic patients 13, which correlates with Th2‐attractant chemokines and disease severity 14.

Importantly, TSLP‐elicited DCs are essential for promoting Th2 cell‐mediated immune responses 15. However, it remains unclear how antigen‐specific type 2 responses are initiated, given that Th2 cell polarization relies on IL‐4 and DCs, whereas naive DCs and CD4^+^ T cells do not produce IL‐4. Prior studies from us and others demonstrated that basophils play a role in modulating type 2 responses by producing IL‐4 after crosslinking with surface Fc receptor for IgE (FcεRI) and by acting as APCs to prime Th2 cell differentiation 16, 17, 18, 19. Consistently, depleting TSLP or removing basophils abrogates airway inflammation in eosinophilic asthma 20, indicating their necessity for the pathogenesis of allergic asthma 21, 22. Although these studies suggest that TSLP and basophils contribute to type 2 responses, less is known about how TSLP ‘educates’ basophils and whether epithelial PRRs affect TSLP production to control innate cell activation.

In this work, we demonstrate that TLR2 expression on airway epithelia were stimulated by various allergens. We also describe an axis linking TLR2‐TSLP to basophil activation during type‐2‐cell‐mediated airway inflammation and further elucidate the underlying molecular signaling pathways.

## Results

### The expression of PRRs, cytokines, and chemokines in the lung following allergen challenge

**Figure 1:**
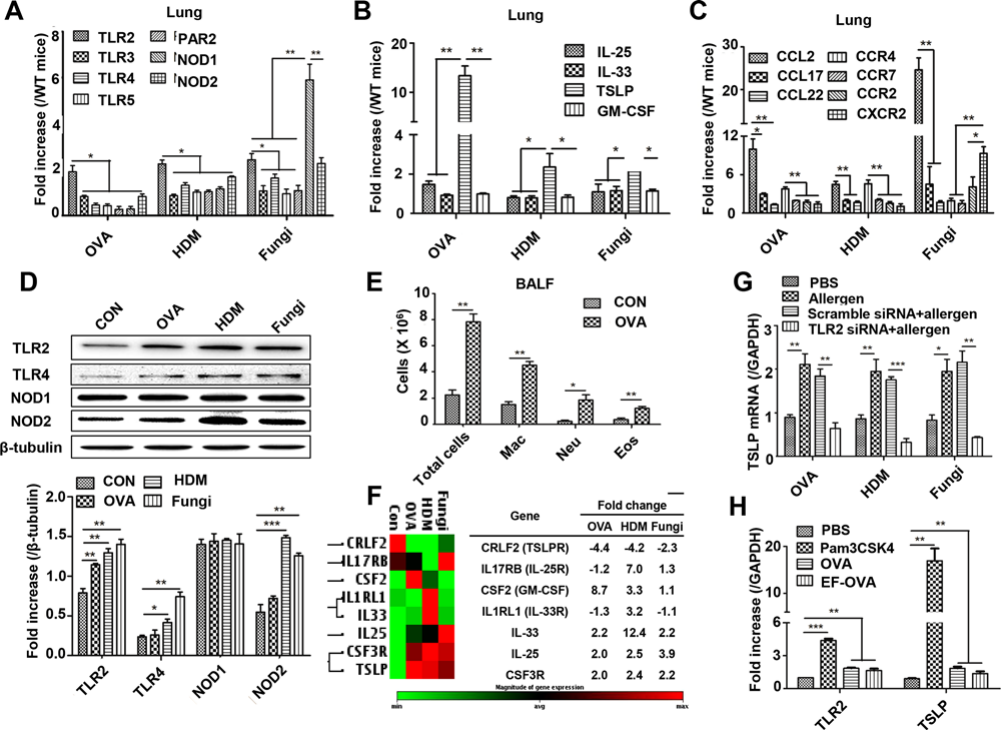
The expression levels of PRRs, cytokines, and chemokines in lung cells following allergen challenge. (A) Expression of PRRs in lung tissues after stimulation of mice with OVA, HDM, and fungi as determined by real‐time‐PCR. (B) Quantification of IL‐25, IL‐33, GM‐CSF, and TSLP mRNA in lung tissues. (C) mRNA quantification of chemokines and their receptors in lung tissues. (D) Expression (top) and gray analysis (bottom) of PRRs in BEAS‐2B cells after stimulation with different allergens was analyzed by western blot. (E) Calculation of cells in BALF. (F) Relative expression of cytokine genes in BEAS‐2B cells with OVA, HDM, and fungi stimulation. (G) Analysis of TSLP mRNA levels in BEAS‐2B cells after TLR2 siRNA treatment. (H) Expression of TLR2 mRNA and TSLP mRNA in BEAS‐2B with Pam3CSK4, OVA, and EF‐OVA. For RT‐PCR and calculation of cells in BALF, data are pooled from three independent experiments with five mice each. For analysis of TLR2 mRNA and TSLP mRNA in vitro, data are pooled from five independent experiments. For PCR Array, data are pooled from three independent experiments and one representative heat map of three independent experiments is shown. One representative western blot of five independent experiments is shown. Values are shown as mean ± SD and statistical analysis employed unpaired two‐tailed Student's *t*‐test. *p < 0.05, **p < 0.01, and ***p<0.001.

To identify the specific PRRs expressed by lung cells in response to allergen challenge, we analyzed several PRRs that were implicated in allergic airway inflammation. Ovalbumin (OVA), HDM, and fungi (*Alternaria alternata*) drastically upregulated TLR2 expression in the lung, although HDM and fungi also somewhat increased TLR4, NOD1, and NOD2 levels as determined by real‐time quantitative PCR (RT‐qPCR) (Fig. 1A).

Epithelial‐derived cytokines, such as IL‐25, IL‐33, TSLP, and granulocyte‐macrophage colony stimulating factor (GM‐CSF), play an important role in mucosal immunity through activation of innate cells 12. Therefore, we determined the production of these cytokines in the lung induced by OVA, HDM, or fungi and found that TSLP mRNA was the highest among these measured cytokines (Fig. 1B). In addition, we analyzed the mRNA levels of chemokines in the lung and found that CCL2 expression was significantly increased though CCR2, CCR4, and CXCR2 expression was also slightly increased (Fig. 1C). Collectively, these results suggest that lung epithelial cells are the dominant populations that expressed TLR2 and secreted TSLP and CCL2 upon allergen stimulation.

Next, we investigated in vitro whether human bronchial ECs have similar functional features such as TLR2‐dependent TSLP production as observed in mice. We stimulated the human bronchial EC line (BEAS‐2B) with OVA, HDM, and fungi to examine the induction of TLR2, TLR4, NOD1, and NOD2. In addition to TLR4 and NOD2, the expression of TLR2 was significantly increased, a result similar to the mouse data (Fig. 1D). In addition, we counted the numbers of total cells, neutrophils, macrophages, and eosinophils and found that total cells also increased (Fig. 1E). We then analyzed the TLR2‐induced cytokine expression profile using PCR Array (Human Allergy & Asthma) and siRNA intervention. We found that these three allergens significantly induced TSLP production compared to the controls (Fig. 1F). At the same time point, TLR2 siRNA significantly decreased allergen‐induced TSLP expression (Fig. 1G). Finally, we used endotoxin‐free OVA (EF‐OVA) to exclude the effect of OVA‐contaminated endotoxin, to stimulate BEAS‐2B. We found that both general‐OVA and EF‐OVA increased TLR2 expression and TSLP production without significant difference between two groups, indicating that OVA but not contaminated endotoxin is attributed to induction of TLR2 expression and subsequent TSLP production. Further study showed that TLR2 and TSLP expression profile from BEAS‐2B was significantly increased by adding Pam3CSK4 (TLR2 agonist) (Fig. 1H). These results suggest that TLR2 is critical for induction of airway epithelial TSLP.

### TLR2 signaling promotes a type 2 immune response to OVA challenge

**Figure 2:**
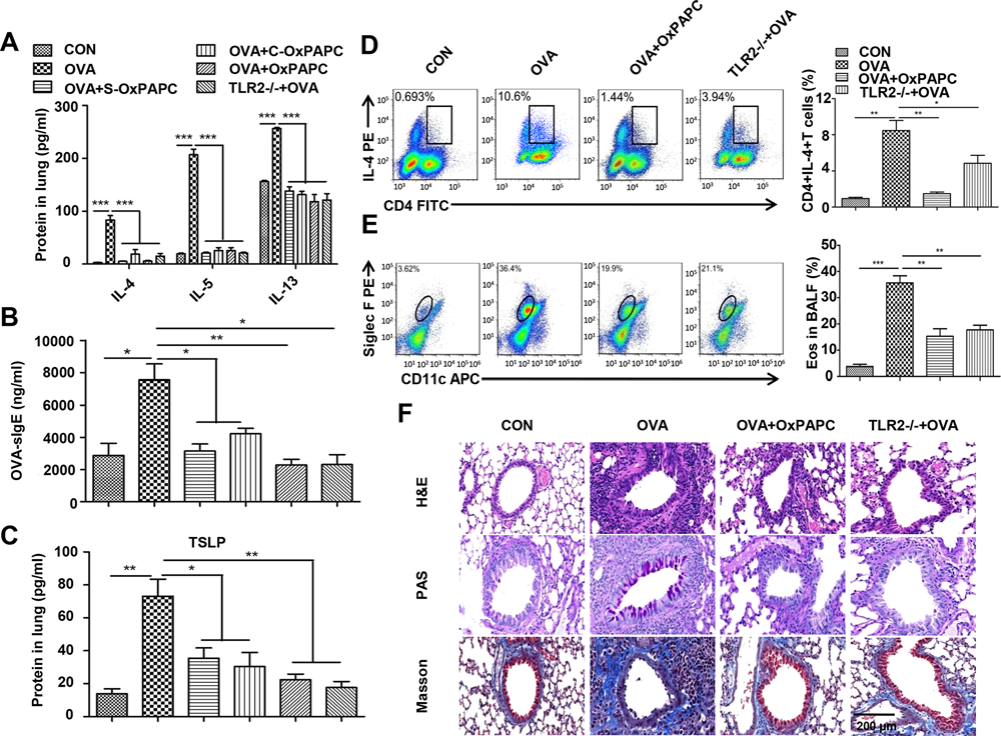
TLR2 signaling induces a type 2 immune response to OVA challenge. OVA‐immunized TLR2^−/−^mice and WT mice treated with OxPAPC in sensitization phase (S‐OxPAPC) or in challenge phase (C‐OxPAPC) or in both phases. (A) Quantification of lung IL‐4, IL‐5, and IL‐13 by ELISA. (B) Quantification of serum OVA‐specific IgE (OVA‐sIgE) by ELISA. (C) Quantification of lung TSLP by ELISA. (D) Proportion of Th2 cells in MLNs was analyzed by flow cytometry. The gating scheme employed is depicted in Supporting Information Figure 1. (E) Proportion of eosinophils (CD11c^−^Siglec F^+^) in BALF. The gating scheme employed is depicted in Supporting Information Figure 2. (F) Lung tissue sections were analyzed by H&E, PAS, and Masson staining. Scale bar represents 200 μm. For ELISA, data are pooled from three independent experiments with five mice each. For flow cytometry in (D) and (E), one representative flow analysis of three independent experiments with five mice each is shown. For statistical analysis of flow cytometry, data are pooled from three independent experiments with five mice each. Values are shown as mean ± SD and statistical analysis employed unpaired two‐tailed Student's *t*‐test. *p < 0.05, **p < 0.01, and ***p < 0.001.

The OVA‐challenged mouse is a classical asthma model. Thus, we performed intranasal administration of OVA to TLR2‐deficient (TLR2^−/−^) mice and WT C57BL/6 mice that were treated with a TLR2 antagonist, oxidized 1‐palmitoyl‐2‐arachidonyl‐*sn*‐glycero‐3‐phosphorylcholine (OxPAPC), to investigate the importance of TLR2 expression on airway lumen in the adaptive immune response to OVA. We found that type 2 cytokines (IL‐4, IL‐5, and IL‐13) in the lungs of OVA‐immunized TLR2^−/−^mice and WT mice treated with OxPAPC were dramatically decreased compared to their respective controls (Fig. 2A). To clarify whether TLR2 signaling is involved only in sensitization phase or in challenge phase, we also administered OxPAPC in sensitization phase (day 0, 7, and 14) (OVA + S‐OxPAPC) and challenge phase (day 23, 24, and 25) (OVA + C‐OxPAPC), respectively. Results showed that blockade of TLR2 either in sensitization phase or in challenge phase decreased levels of IL‐4, IL‐5, and IL‐13 in the lung (Fig. 2A). In addition, levels of OVA‐specific IgE (Fig. 2B) and TSLP (Fig. 2C) were significantly decreased in both OVA + S‐OxPAPCand OVA + C‐OxPAPC groups. However, no differences in IL‐4, IL‐5, IL‐13, and OVA‐sIgE were observed among OVA + OxPAPC, OVA + S‐OxPAPC, and OVA + C‐OxPAPC groups. These results suggest that TLR2 signaling is involved in both sensitization phase and challenge phase.

Next, we isolated mediastinal LN (MLN) cells from TLR2^−/−^ and OxPAPC‐treated WT mice and challenged them with OVA in vitro for 5 consecutive days to evaluate Th2 cell differentiation. The percentage of CD4^+^IL‐4^+^ cells from both TLR2^−/−^ and OxPAPC‐treated WT mice was significantly lower than that from OVA‐treated WT mice (Fig. 2D), indicating that the TLR2 signaling strength controls Th2 cell differentiation. Furthermore, blocking TLR2 decreased the number of eosinophils in BALF (Fig. 2E) and dampened peribronchial infiltration, mucus secretion, and collagen deposition in both TLR2^−/−^ and OxPAPC‐treated WT mice (Fig. 2F). Together, these results strongly indicate that TLR2 signaling mediates a Th2‐driven immune response to OVA.

### Effect of TLR2 signaling on innate immune cells in the lung

**Figure 3:**
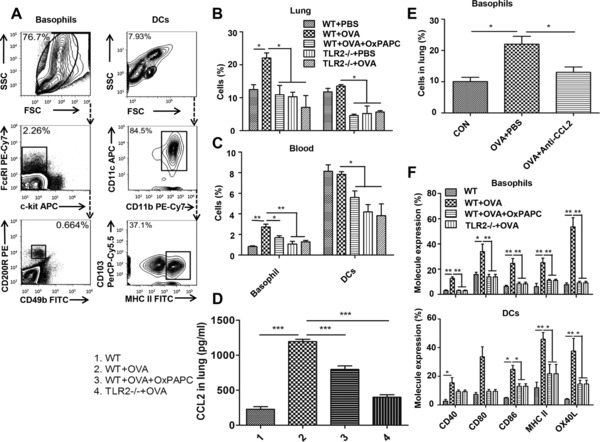
The effect of TLR2 signaling on innate immune cells in the lung. OxPAPC‐treated mice or TLR2^−/−^ mice received OVA immunization. Lung cell suspension was subjected to flow cytometry analysis for the number and molecule expression of basophils and DCs in the lung. Alternatively, lung homogenates were subjected to protein analysis by ELISA. Gating strategy for basophils and DCs in blood by flow cytometry was shown in Supporting Information Figure 3. (A) Gating strategy for basophils and DCs in lung. (B) Quantification of lung c‐kit^−^ CD49b^+^ FcεRIa^+^ CD200R^+^ basophils and CD11c^+^ CD11b^+^ MHCII^+^ CD103^−^ DCs in OVA‐immunized TLR2^−/−^ and OxPAPC‐treated WT mice. (C) Quantification of basophils and DCsin peripheral blood. (D) Quantification of lung CCL2. (E) Determination of basophils in the lung after CCL2 blockade. (F) Expression levels of co‐stimulatory molecules on basophils and DCs in the lung. For flow cytometry, one representative flow analysis for basophils and DCs of three independent experiments with five mice each is shown. For statistical analysis of flow cytometry and ELISA, data are pooled from three independent experiments with five mice each. Values are shown as mean ± SD and statistical analysis employed unpaired two‐tailed Student's *t*‐test.*p < 0.05, **p < 0.01, and ***p < 0.001.

To determine how TLR2 signaling affects basophils and DCs, we examined the contribution of TLR2 to the influx of innate immune cells into the lung by administration of OVA to TLR2^−/−^mice and WT mice treated with or without OxPAPC. Basophils and DCs in the lung were identified as c‐kit^−^ CD49b^+^ FcεRIa^+^ CD200R^+^ cells and CD11c^+^ CD11b^+^ MHCII^+^ CD103^−^ cells, respectively (Fig. 3A). The number of lung basophils in OVA‐immunized mice was significantly higher than that in TLR2^−/−^ mice and OxPAPC‐treated WT mice (Fig. 3B). Interestingly, the number of lung DCs did not increase in OVA‐immunized mice compared to WT controls but significantly decreased in both TLR2^−/−^ mice and OxPAPC‐treated WT mice (Fig. 3B).

We proposed that the alteration of lung basophils was regulated by the chemokine CCL2, which might accelerate the release of basophils from BM into the blood stream and recruitment of blood basophils into the lung. Therefore, we first analyzed the number of basophils in peripheral blood from mice stimulated by OVA with or without OxPAPC treatment and found that the number of basophils was significantly higher after OVA immunization, which is consistent with the change in lung CCL2 levels (Fig. 3C). Similarly, the proportion of DCs in peripheral blood was not increased in the OVA immunization group, consistent with that in the lung. However, TLR2^−/−^ mice and OxPAPC‐treated mice exhibited a reduced number of basophils and DCs in the blood (Fig. 3C).

We next investigated whether TLR2 signaling determines CCL2 production. Our results showed that CCL2 production was significantly higher in OVA‐treated mice but was significantly lower in both TLR2^−/−^ mice and OxPAPC‐treated WT mice compared to their controls (Fig. 3D).To further verify recruitment of basophils into the lung through CCL2, mice were intranasally treated with a CCL2 antibody during the challenge phase. The results demonstrated that blockade of CCL2 resulted in decreased number of basophils in the lung, indicating a critical role of CCL2 in the recruitment of basophils into the lung (Fig. 3E).

Finally, we evaluated the maturation of lung basophils and DCs by assessing the expression of their surface markers. The expression of co‐stimulatory molecules (CD40, CD80, CD86, MHC II, and OX40L) was significantly increased, but was abrogated in TLR2^−/−^ mice and OxPAPC‐treated mice (Fig. 3F). Therefore, our data indicate that allergen‐induced TLR2 increased CCL2 production, recruited basophils but not DCs into the lung, and did not alter the maturation of basophils and DCs.

### TLR2 signaling promotes TSLP production and activates basophils and DCs to drive airway inflammation

**Figure 4:**
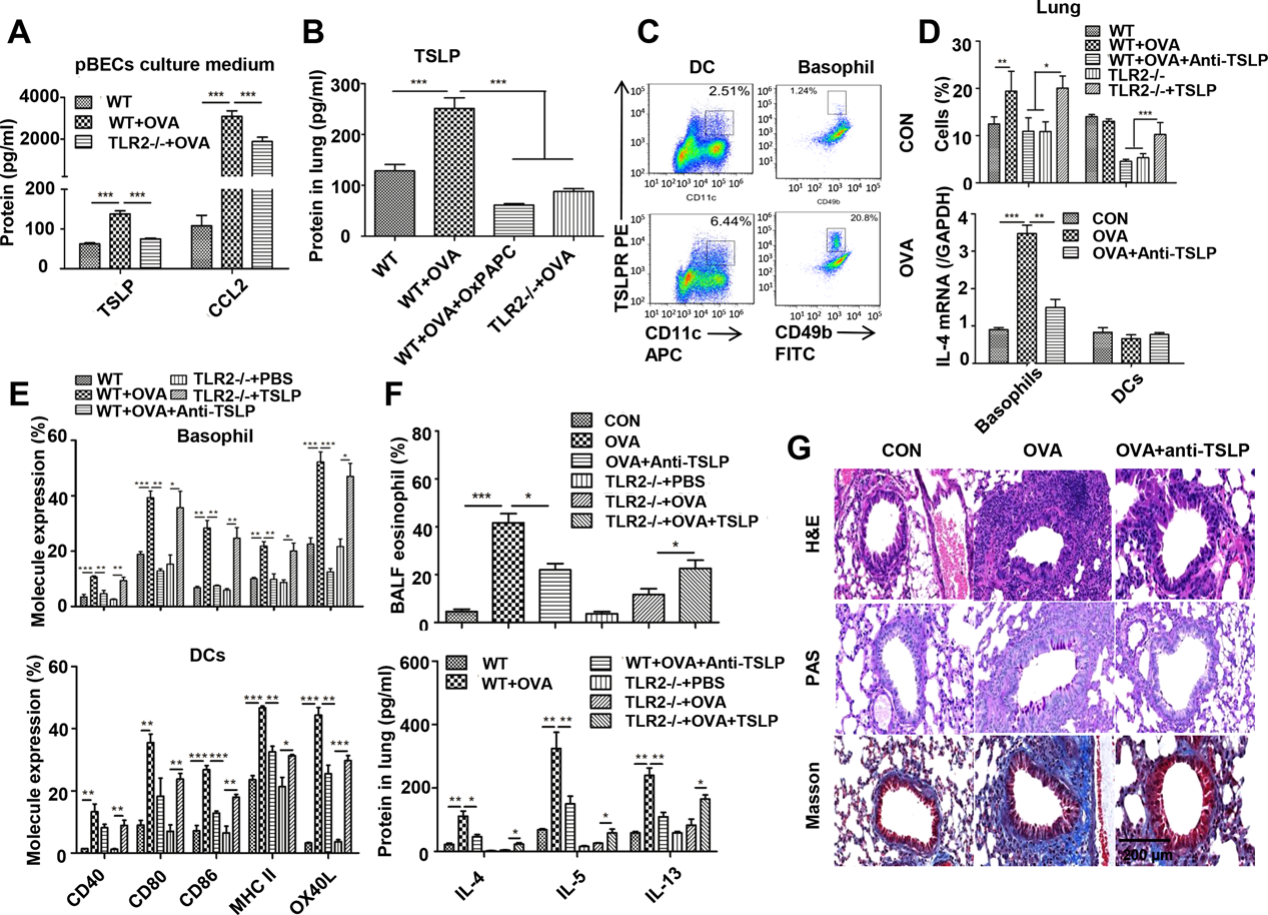
TLR2 signaling promotes TSLP production to activate basophils and DCs leading to airway inflammation. For in vivo study, TLR2^−/−^ mice or OxPAPC‐treated WT mice received OVA sensitization and OVA challenge. Lung cells were subjected to flow cytometry analysis for determination of the number and molecule expression of basophils and DCs. Alternatively, lung cells were subjected to basophil or DC isolation by flow cytometry followed by RNA extraction for RT‐PCR. For some experiments, lung homogenates were used to determine protein levels by ELISA or histology. Gating strategy for basophils and DCs in lung by flow cytometry was shown in Supporting Information Figure 3. (A) Quantification of TSLP and CCL2 in the culture supernatant of pBECs by ELISA. pEBCs were isolated from WT or TLR2^−/−^ mice followed by stimulation with OVA. Culture supernatant was collected for determination of cytokines. (B) Quantification of the lung TSLP level was determined by ELISA. (C) Expression of TSLPR on lung basophils and DCs was analyzed by flow cytometry. (D) Quantification of the number and IL‐4 mRNA expression of lung basophils and DCs by qPCR. (E) Expression of co‐stimulatory molecules on lung basophils and DCs by flow cytometry. (F) Determination of eosinophils in BALF by flow cytometry and quantification of IL‐4, IL‐5, and IL‐13 in lung homogenates by ELISA. (G) Histological analysis of lung tissues by H&E, PAS, and Masson staining. Scale bar represents 200 μm. For ELISA, RT‐PCR, calculation of cells in BALF, and flow cytometry, data are pooled from three independent experiments with five mice each. One representative flow analysis for TSLP receptor expression on basophils of three independent experiments with five mice each is shown. Values are shown as mean ± SD and statistical analysis employed unpaired two‐tailed Student's *t*‐test.*p < 0.05, **p < 0.01, and ***p < 0.001.

Since TSLP is an efficient pro‐inflammatory cytokine that plays a key role in the activation of innate immune cells and the induction of adaptive immunity, we examined TLR2‐dependent TSLP expression in primary bronchial epithelial cells (pBECs) isolated from TLR2^−/−^ mice. OVA stimulation increased the level of TSLP protein in the culture supernatant of pBECs from OVA‐stimulated WT mice but failed to increase its production of pBECs from TLR2^−/−^ mice, accompanying CCL2 changes (Fig. 4A). Next, we detected lung TSLP in allergic mouse models and found an elevated level in the OVA immunization group, but this increase was abolished in both OVA‐immunized TLR2^−/−^ mice and OxPAPC‐treated WT mice (Fig. 4B). We subsequently determined the expression of TSLP receptor (TSLPR) on lung basophils and DCs and noticed that OVA immunization significantly increased TSLPR on both basophils and DCs (Fig. 4C), consistent with increased TSLP production in the lung. The relationship between TSLP and its receptor undoubtedly indicates an indispensable role of TSLP in activating basophils and DCs to prime mucosal immunity.

We further investigated the role of TSLP in mouse models of airway inflammation through intranasal instillation of TSLP antibodies and phosphate buffer saline (PBS) as a control. Interestingly, antibody neutralization of TSLP significantly decreased the number of lung basophils and DCs of OVA‐immunized mice (Fig. 4D). Since initial IL‐4 production is critical for Th2 cell differentiation, we determined IL‐4 mRNA in lung basophils and DCs and found that IL‐4 mRNA expression in basophils rather than DCs, was increased after OVA immunization, but was rescued after airway TSLP neutralization (Fig. 4D). To determine whether TSLP affects phenotypic maturation of basophils and DCs, we examined the expression of co‐stimulatory molecules in airway basophils and DCs and observed that the expression of CD40, CD80, CD86, MHC II, and OX40L in OVA‐immunized mice was significantly increased compared to control mice, but this response was selectively abolished in OVA + Anti‐TSLP mice (Fig 4E). To verify the TSLP effects on basophils and DCs in the lung and co‐stimulatory molecule expression on these cells, TLR2^−/−^ mice were intranasally administered with recombinant mouse TSLP at days 23, 24, and 25 and PBS as a control. The results demonstrated that administration of TSLP could increase numbers of basophils and DCs (Fig. 4D), and these molecule expressions on two cells compared to PBS‐treated control (Fig 4E). We also measured the levels of type 2 cytokines and eosinophils in the BALF and noticed that anti‐TSLP antibody decreased levels of IL‐4, IL‐5, and IL‐13 in lungs and reduced numbers of eosinophils in BALF of OVA‐induced mice. However, administration of TSLP protein in OVA‐stimulated TLR2^−/−^ mice markedly increased the levels of type 2 cytokines in the lung and the number of eosinophils in BALF when compared to PBS‐ or OVA‐treated mice (Fig. 4F). These results indicate that TLR2‐induced TSLP directly regulates activation of basophils and DCs as well as type 2 responses.

In addition, lung tissue histology analysis demonstrated that inflammatory responses were decreased in the OVA + Anti‐TSLP group compared with the OVA group (Fig. 4G). These results show that allergen‐induced TLR2 overexpression regulates production of TSLP, which participates in the airway inflammatory response.

### TLR2–NF‐κB/JNK signaling pathways mediate TSLP production to prevent apoptosis of basophils and DCs

**Figure 5:**
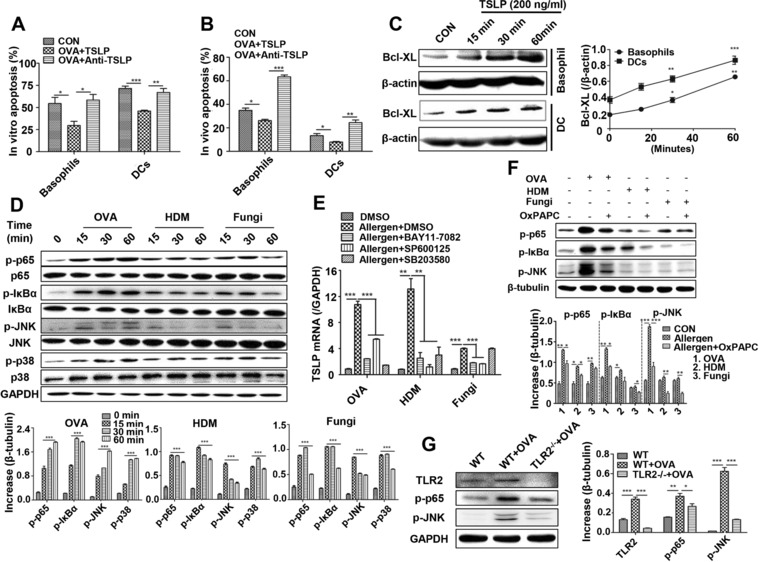
TLR2–NF‐KB/JNK signaling pathways mediate TSLP production to prevent apoptosis of basophils and DCs. (A) OVA‐immunized mice received TSLP or anti‐TSLP were subjected to flow cytometry for determination of apoptosis in basophils and DCs in the lung. (B) Lung cell suspensions of OVA‐immunized mice were stimulated with TSLP or anti‐TSLP in vitro and were subsequently subjected to flow cytometry for the analysis of apoptosis in basophils and DCs. (C) Expression level of anti‐apoptosis protein Bcl‐XL in bone marrow‐derived basophils (BMBas) and BMDCs after TSLP treatment at different time points. (D) Analysis of p‐p65, p‐JNK, and p‐p38 in BEAS‐2B cells after OVA, HDM, and fungi stimulation in BEAS‐2B cells by western blot. (E) Expression level of TSLP mRNA in BEAS‐2B cells treated with inhibitors of NF‐κB, JNK, and p38 followed by OVA, HDM, and fungi stimulation by RT‐PCR. (F) Analysis of NF‐κB and p‐JNK in OxPAPC‐pretreated BEAS‐2B cells with OVA, HDM, and fungi stimulation by western blot. (G) Analysis of NF‐κB and JNK signals in OVA‐stimulated pBECs from WT or TLR2^−/−^ mice by western blot. For statistical analysis of apoptosis, data are pooled from three independent experiments with five mice each. For RT‐PCR and western blot, data are pooled from five independent experiments. One representative western blot of five independent experiments is shown. Values are shown as mean ± SD and statistical analysis employed unpaired two‐tailed Student's *t*‐test. *p < 0.05, **p < 0.01, and ***p < 0.001.

The results above have demonstrated that TSLP is critical to the number and activation of basophils and DCs. Thus, we proposed that TSLP may prolong the survival of basophils and DCs to promote the inflammatory response. Therefore, we examined the effect of TSLP on the apoptosis of basophils and DCs. First, we determined whether TSLP affects the apoptosis of lung basophils and DCs in vitro. Lung cells from OVA‐immunized mice were cultured in the presence of recombinant mouse TSLP or anti‐TSLP antibody for 24 h. TSLP stimulation resulted in significant reduction in basophil and DC apoptosis, whereas anti‐TSLP antibody greatly promoted basophil and DC apoptosis (Fig. 5A). We further analyzed OVA‐immunized mice after treatment with TSLP or anti‐TSLP antibody and observed similar results to those found in vitro (Fig. 5B), with the exception of a mild change detected in basophil and DC apoptosis in the control and OVA + TSLP groups. To verify these findings, the levels of the anti‐apoptotic protein Bcl‐XL in BM‐derived basophils and BM‐derived DCs were also examined. TSLP treatment significantly increased Bcl‐XL expression levels (Fig. 5C), indicating that TSLP is critical for preventing apoptosis of activated basophils and DCs.

Three signaling pathways, PI3K/Akt, MAPK, and NF‐κB, have been reported to be involved in TSLP production 23. We therefore evaluated the activation of these axes in BEAS‐2B cells in response to three allergens. We found higher levels of p65, JNK, and p38 phosphorylation after 15 min of stimulation, lasting for 1 h after stimulation. Similarly, there was an increased level of p‐IκBα, suggesting a weak capability to suppress p65 (Fig. 5D). Next, to elucidate whether the NF‐κB, JNK, and p38 signaling pathways were critical for TSLP production, BEAS‐2B cells were pretreated with small molecule inhibitors, BAY11‐7082, SP600125, and SB203580, to inhibit NF‐κB, JNK, and p38, respectively. OVA‐, HDM‐, or fungi‐induced TSLP production was associated with activation of the NF‐κB and JNK signaling pathways (Fig. 5E). However, p38 signaling did not alter fungi‐induced TSLP production (Fig. 5E). Surprisingly, the increased phosphorylation of p65 and JNK was strikingly reversed when BEAS‐2B cells were pretreated with TLR2 inhibitor OxPAPC (Fig. 5F). Finally, we determined the phosphorylation levels of p65 and JNK in OVA‐stimulated pBECs from TLR2^−/−^ mice. We found that phosphorylation of p65 and JNK was significantly decreased in pBECs from TLR2^−/−^ + OVA mice (Fig. 5G). Altogether, these results demonstrate that human bronchial ECs share functional features of mouse bronchial ECs and that the NF‐κB and JNK signaling pathways participate in epithelial TLR2‐derived production of TSLP.

### TLR2‐derived TSLP activates basophils and DCs through NF‐κB and JNK signaling in vivo

**Figure 6:**
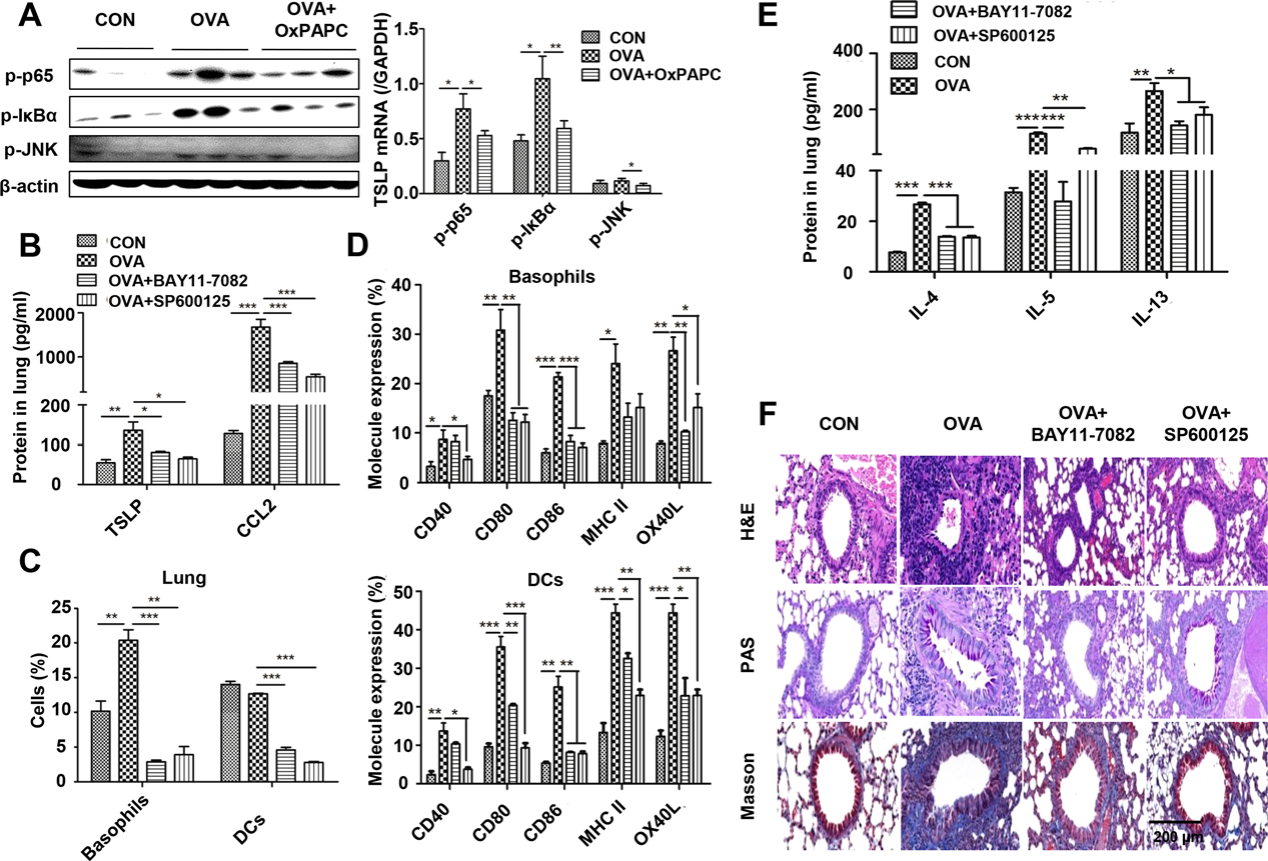
NF‐κB and JNK signaling pathways participate in TLR2‐derived TSLP production and activation of basophil and DC in vivo. OVA‐immunized mice were intraperitoneally injected with BAY11‐7082 or SP600125 to inhibit the NF‐κB or JNK pathway, respectively. (A) Analysis of p‐p65, p‐IКBα, and p‐JNK in the lung by western blot. (B) Quantification of TSLP and CCL2 in the lung by ELISA. (C) Number of basophils and DCs in the lung by flow cytometry. (D) Expression levels of co‐stimulatory molecules on basophils and DCs in the lung analyzed by flow cytometry. (E) Quantification of IL‐4, IL‐5, and IL‐13 in the lung by ELISA. (F) Histological analysis of lung tissues by H&E, PAS, and Masson staining. Scale bar represents 200 μm. For statistical analysis of western blot, data are pooled from three independent experiments with five mice each and one representative western blot is shown. For statistical analysis of ELISA and flow cytometry, data are pooled from three independent experiments with five mice each. Values are shown as mean ± SD and statistical analysis employed unpaired two‐tailed Student's *t*‐test. *p < 0.05, **p < 0.01, and ***p < 0.001.

To determine whether TLR2 is required to activate NF‐κB or JNK signaling in vivo, we treated OVA‐immunized WT mice with or without OxPAPC to analyze activation of NF‐κB and JNK signaling. Compared to OVA immunization alone, blocking TLR2 with OxPAPC decreased phosphorylation of p65, IκBα, and JNK (Fig. 6A), indicating that the NF‐κB and JNK signaling pathways mediate TLR2‐derived TSLP production. To validate the role of NF‐κB and JNK activation in the production of TSLP, we intraperitoneally administered small chemical inhibitors to OVA‐immunized mice and found greatly reduced TSLP and CCL2 in the lung after inhibition of either the NF‐κB or JNK signaling pathway (Fig. 6B), with a consistently significant decrease in basophils and DCs in the lung (Fig. 6C). Next, we examined whether inhibition of NF‐κB or JNK signaling affects the activation status of basophils and DCs. Compared to the OVA group, inhibition of NF‐κB and JNK signaling almost abrogated the increased expression of CD40, CD80, CD86, MHC II, and OX40L (Fig. 6D). Furthermore, the levels of type 2 cytokines (IL‐4, IL‐5, and IL‐13) were significantly decreased following inhibition of either the NF‐κB or JNK signaling pathway (Fig. 6E), and histological analysis results showed decreased inflammatory responses in the lung (Fig. 6F). These results indicate that both NF‐κB and JNK signaling pathways are involved in the activation of basophils and DCs. Collectively, epithelial TLR2–NF‐κB/JNK–TSLP signaling recruits basophils and activates resident DCs in the lung, leading to type 2 immune responses.

## Discussion

Several reports have indicated the roles of TLR2 signaling in OVA‐induced allergic airway inflammation 24, 25, 26, leaving the roles of airway ECs to be studied. Herein, we demonstrate that airway epithelial TLR2–NF‐κB/JNK–TSLP axis plays an important role in the initiation of allergic inflammation. TLR2 is not the only TLR that participates in pathogen sensing, but may be especially critical in the initial stages of the innate immunity response. TLR2 activation has been reported to cause initiation of both type 1 and type 2 responses 27, 28, 29. A potential explanation for the ability of TLR2 signaling to activate both pro‐ and anti‐inflammatory responses is that the outcome depends on the doses, nature of TLR2 agonists, and genetic background of the host 30. In this study, intranasal blockade of the TLR2 route was designed to influence the airway epithelial response and to avoid a potential systemic effect on other cells. We further verified these effects by isolation of pBECs from TLR2^−/−^ mice and demonstrated that ligation of TLR2 by allergen was indeed proinflammatory, at least by promoting TSLP production.

HDM and fungi are clinically relevant allergens that seem to contain TLR2 ligand, such as LPS. An interesting finding is that OVA can also activate bronchial ECs through TLR2. Explanation of the fact that commercial OVA activates TLR2 is attributed to the contamination of LPS 31. However, depletion of LPS from OVA did not affect the OVA‐induced influx of eosinophils and lymphocytes to airways and increase of Th1 and Th2 cytokine in lung tissues 32, which is consistent with our results. Furthermore, either using of commercial OVA or EF‐OVA increased TLR2 or TSLP of airway ECs with no significant difference between OVA and EF‐OVA groups. These findings suggest that it was OVA but not contaminated endotoxin (or LPS) stimulated TLR2 signaling. It was reported that OVA contains a structure of serpin, a protease inhibitor playing essential roles in coagulation and inflammation 33. In contrast, non‐inhibitory extracellular serpins also perform a wide array of important roles 34, 35. The non‐inhibitory serpin OVA is the most abundant protein in egg white that is a common allergen of food allergen‐induced asthma 36. Whether the interaction of non‐inhibitory serpin OVA with protease activates TLR2 needs to be further investigated.

The decreased numbers of lung basophils and DCs after intranasal TLR2 antagonist treatment most likely result from decreased chemokines and increased apoptosis 37. Consistently, we observed an increase in CCL2 in the lung of asthmatic mice model, with a simultaneous increase in CCR2. Nevertheless, CCL2 was sharply decreased after intranasal administration of TLR2 antagonist, which was followed by decreased number of basophils and DCs in the lung. Furthermore, airway blockade of CCL2 decreased number of basophils in the lung, suggesting a CCL2‐mediated chemotaxis. In addition, unlike the fixed number of resident DCs in airway tissues, OVA immunization led to a significant influx of basophils into the lung, which was likely recruited from the BM via blood transport. Thus, we may conclude that TLR2 signaling promotes CCL2 production, which recruits basophils into the lung.

Another paradigmatic feature of epithelial TLR activation is its potential to produce a large number of cytokines. Using mRNA expression analysis in vitro, we observed a rapid production of TSLP in a TLR2‐dependent manner. Once secreted into the airways, TSLP primes basophils and DCs to induce T cell differentiation in areas of draining MLNs 38. This behavior depends on high levels of TSLP, as blocking TSLP in the airway lumen strongly reduced the number of basophils and DCs in the lung and decreased type 2 immune responses. When basophils are recruited into the lung, recruited basophils and resident DCs are activated by TSLP, uptake antigens, and migrate to lymph nodes to induce T cell differentiation, thereby fulfilling their maturation program 39. Consistently, we found that increased expression of co‐stimulatory molecules on lung basophils and DCs was dramatically reversed by TSLP antibody or TLR2 deficiency, which was rescued by intranasal administration of recombinant TSLP. This maturation behavior closely coincides with their role in the induction of type 2 responses, as we and others have previously reported 40, 41. Blockade of airway TSLP decreased IL‐4 production in lung basophils, suggesting its critical role in the induction of Th2 response through triggering initial IL‐4 synthesis. In addition, TSLP prolongs the survival of basophils and DCs by increasing anti‐apoptotic Bcl‐XL, which is consistent with a role in promoting survival of inflammatory cells as previously reported 42. Of note, it cannot exclude the possibility that TSLP ensures the number of basophils and DCs by promoting their expansion. These results indicate a strong function of TSLP in activating mucosal immune responses in allergic airway inflammation.

Lastly, we elucidated that TSLP production induced by three different allergens depends on NF‐κB and JNK activation and further verified in vivo by injecting NF‐κB and JNK inhibitors. Other PRRs, such as NODs and TLR4, also signal through NF‐κB and MAPK activation 43. Similarly, we found that TLR4 and NOD2 levels were also increased by HDM and fungi stimulation, respectively. To rule out the effect of OxPAPC on TLR4 and to substantiate the function of TLR2, we utilized pBECs from control TLR2^−/−^ mice and asthmatic TLR2^−/−^ mice and found similar results to that in cells with OxPAPC. How TLR4 and NOD2 are activated on ECs is a matter of intense study, and our data provide a potential target in the innate immune response to allergens.

In summary, these results place TLR2 at an important position of immune network. Modulation of TLR2‐mediated signaling might therefore provide novel directions for intervention within a broad spectrum of immune diseases.

## Materials and methods

### Allergen‐induced airway inflammation

TLR2^−/−^(Model Animal Research Center of Nanjing University) and WT C57BL/6 mice (Shanghai Laboratory Animal Co., Ltd, China) at 6–8 weeks of age were utilized in this study and were housed in the specified‐pathogen‐free (SPF) facilities in the Research Center for Experimental Medicine of Ruijin Hospital affiliated with Shanghai Jiao Tong University School of Medicine. All animal experiments were approved by the Ruijin Hospital Animal Ethics Committee. The asthmatic mouse model induced by OVA (Sigma–Aldrich, St Louis, MO) or EF‐OVA (endotoxin concentration < 0.1 EU/mg, EndoGrade Ovalbumin; Hyglos, Regensburg, Germany) was performed using previously described methods 44. In the same experiments, OVA‐sensitized and OVA‐challenged mice were dosed intranasally with 75 μg/50 μL TLR2 antagonist OxPAPC (Invivogen, San Diego, CA, USA) on days 0, 7, and 14; 7.5 mg/kg recombinant mouse TSLP on days 23, 24, and 25; TSLP‐mAb (R&D Systems, Minnesota, USA) on days 23, 24, and 25; 100 μg/50 μl CCL2 45 on days 0, 7, 14, 23, and 24, or were given intraperitoneal injections of 0.6 mg BAY11‐7082 (an NF‐κB inhibitor) or 0.9 mg SP600125 (a JNK inhibitor, Selleckchem, Houston, Texas, USA) dissolved in DMSO (Sigma–Aldrich, St. Louis, Missouri, USA), and diluted with corn oil (Sigma–Aldrich) on days 1, 13, and 22. For HDM or fungi‐induced asthma models, mice were dosed intranasally with 50 μg/ml HDM (Greer Laboratories, Lenoir, NC, USA) or 50 μg/ml fungi (*Alternaria alternata*, Greer Laboratories) at days 0, 7, 14, 15, and 16 and were sacrificed on day 17.

### Bronchoalveolar lavage

Bronchoalveolar lavage was performed as previously described 32. BAL cytospin slides were prepared with 6 × 10^4^ cells/slide (Cytospin4; Shandon, Pittsburgh, PA, USA) and stained with Diff‐Quik Stain Set. Total BAL cell numbers were counted, and cells were identified as macrophages, eosinophils, neutrophils, and lymphocytes from a count of at least 400 cells.

### Tissue harvest and preparation of single‐cell suspensions

After 24 h of the final allergen challenge, mice were sacrificed by CO_2_ inhalation. Blood samples were collected for DC and basophil counts. A single cell suspension was obtained as we previously described 41. For some experiments, lung single cell suspensions were stimulated with 100 ng/mL recombinant mouse TSLP or anti‐TSLP antibody for 24 h and were subjected to flow cytometric analysis. MLN cells were plated in 24‐well plates at a density of 5 × 10^6^ cells/mL and stimulated with 500 μg/mL OVA for 5 days, followed by flow cytometric analysis.

### Cell isolation, culture, treatment, and transfection

Isolation of tracheal cells from TLR2^−/−^ and WT C57BL/6 mice was performed as previously described to isolate primary bronchial epithelial cells (pBECs) 46. BEAS‐2B (American Type Culture Collection) cells were incubated for 6 or 24 h with 1 μg/mL Pam3CSK4 47, 500 μg/mL OVA, 500 μg/mL EF‐OVA, 50 μg/mL HDM, and 50 μg/mL Alternaria extract, separately. Cell culture supernatants and cell lysates were collected for detection of protein and mRNA. To perform the transfection assay, BEAS‐2B cells were transfected with 5 nmol/L siRNA against TLR2 or control siRNA using HiPerFect transfection reagent (Qiagen) for 24 h, followed by stimulation with the same concentrations of OVA, HDM, and Alternaria extract, separately. Alternatively, BEAS‐2B cells were pretreated with 1.5 μg/mL OxPAPC for 24 h, followed by stimulation with the same concentrations of OVA, HDM, and fungi, separately. Lastly, pBECs from WT mice or TLR2^−/−^ mice were stimulated with 500 μg/mL OVA for 1 or 24 h. Various signaling molecules from cell lysates and the production of TSLP and CCL2 in culture supernatant were determined by western blot and ELISA.

### Enzyme‐linked immunosorbent assay

Cell culture supernatants, lung homogenates, and serum were prepared as previously reported. The levels of IL‐4, IL‐5, IL‐13, TSLP, and CCL2 were measured with sandwich ELISA kits (Biolegend, San Diego, California, USA) according to the manufacturer's instructions.

### Real time‐PCR

Right lung lobes were flash frozen and homogenized for detection of protein and mRNA. Total mRNA was isolated using the RNeasy kit (Qiagen, Valencia, CA). Real time PCR (RT‐PCR) was performed to assess gene expression of TLR2, TLR3, TLR4, TLR5, NOD1, NOD2, PAR2, IL‐25, IL‐33, TSLP, GM‐CSF, CCL2, CCL17, CCL22, CCR2, CCR4, CXCR2, and CCR7 using SYBR Green (Qiagen, Valencia, CA). Primer sequences were shown in Table 1.

**Table 1:** Primer sequences

	Forward (5′–3′)	Reverse (5′–3′)
mTLR2	AGCCCATTGAGAGGAAAGCC	CCAAAACACTTCCTGCTGGC
mTLR3	GAACAACGCCCAACTGAACC	GAGAAAGTGCTCTCGCTGGT
mTLR4	TCAGAGCCGTTGGTGTATCTT	CCTCAGCAGGGACTTCTCAA
mTLR5	ACTTCGGCTGTTTTCCTGTG	GGAGGCTGTGAATCTGGTTG
mNOD1	TGCTGCTGGAGGAGACCTAT	AAGACAGTCTCGCCATGCTC
mNOD2	ACTCTGTGGGAGATGTTGGAG	TGAGTTTGTTGTTGAAGAGAGCTA
mPAR2	ACGTCTACGCCCTCTACCTT	GTTTCTGGCGTGATCCCTGA
mIL‐25	CGTCCCACTTTACCACAACC	ACACACACACAAGCCAAGGA
mIL‐33	AGTCTCCTGCCTCCCTGAGT	CCTGGTCTTGCTCTTGGTCT
mTSLP	TGAAAGGGGCTAAGTTCGAG	AAATGTTTTGTCGGGGAGTG
mGM‐CSF	TGGTCTACAGCCTCTCAGCA	GACGACTTCTACCTCTTCATTCAAC
mCCL2	TCTGTGCTGACCCCAAGAA	TGTGGAAAAGGTAGTGGATGC
mCCL17	CGAGAGTGCTGCCTGGATTA	TGTTTGTCTTTGGGGTCTGC
mCCL22	CTGATGCAGGTCCCTATGGT	GCAGGATTTTGAGGTCCAGA
mCCR2	CTCAGTTCATCCACGGCATA	TGACAAGGCTCACCATCATC
mCCR4	ATTTGCTGTTCGTCCTGTCC	GAAGATGCCGCTGTAGAAGC
mCXCR2	TGTTCTGCTACGGGTTCACA	AGGAAGACAAGGACGACAGC
mCCR7	AACGGGCTGGTGATACTGAC	AGGACTTGGCTTCGCTGTAG
hTSLP	CCAGAGCCTAACCTTCAATCC	GTTGTGACTTTCCTTTTTCTCCTC

“m” means mouse; “h” means human.

### Western blot analysis

Western blot was performed as previously described 48. Membranes were blotted with antibodies to TLR2, TLR4, NOD1, NOD2 (Santa Cruz Biotechnology, Inc.), p‐p65, p65, p‐JNK, JNK, p‐p38, p38, p‐IκBα, IκBα, Bcl‐XL, β‐actin, β‐tubulin, and GAPDH (Cell Signaling Technology, Danvers, Massachusetts, USA).

### Flow cytometry

Basophils and DCs in peripheral blood, lung and Th2 cells in MLNs were isolated and then washed prior to staining. Cells were blocked with anti‐FcγRII/III (eBioscience, San Diego, CA, USA) at a dilution of 1:200 and stained with fluorescently tagged rat anti‐mouse IgG for the following cell surface antibodies, FcεRIa PE‐Cy7, c‐kit APC, CD49b FITC, CD200R PE, TSLPR BV421(Biolegend, San Diego, California, USA), or stained with CD11c APC, MHC II FITC, CD11b PE‐Cy7, CD103 PerCP‐Cy5.5, and TSLPR BV421. Cells isolated from BALF were stained with Siglec F PE and CD11c APC. Single cell suspensions from MLNs after restimulation with 500 μg/mL OVA in vitro were collected and stained with CD4 FITC and IL‐4 PE. For analysis of apoptosis, lung cells were stained with antibodies listed above and Annexin V FITC and 7‐AAD (BD Pharmingen, San Diego, California, USA). Intracellular IL‐4 staining was performed as previously reported 41. Briefly, cells were stimulated by 20 ng/mL PMA (Sigma–Aldrich) and 1 μg/mL ionomycin (Sigma–Aldrich) for further 6 h and were stimulated with Brefeldin A (eBioscience) at the ratio of 1:1000 for further 2 h. The collected cells were surface stained with CD4 FITC and IL‐4 PE (BD Pharmingen).

### Histological analysis

The right lower lung was isolated and fixed in 4% buffered formalin. Lung sections of 5 μm were stained with H&E, PAS, and Masson to assess inflammation.

### Small molecule inhibitor interference in BEAS‐2B cells

BEAS‐2B cells were seeded at a low density of 1 × 10^5^ for 16–24 h to reach a confluence of 30–50%. Cells were treated for 1 h with one of the following specific pathway inhibitors: 1 μmol/L BAY11‐7082, 10 μmol/L SP600125, or 20 μmol/L SB203580 (a p38 inhibitor). All inhibitors were from Selleckchem. Cells were then stimulated with the same concentrations of OVA, HDM, and fungi, separately. Expression of TSLP was examined by RT‐PCR.

### Statistical analysis

For all experiments, we calculated the differences between groups using unpaired two‐tailed Student's *t*‐test to perform statistical analysis of all data (GraphPad Prism version 5.0; GraphPad Software). Differences were considered to be significant when *p *< 0.05.

## Conflict of interest

The authors declare no financial or commercial conflict of interest.

**Abbreviations**
APCs: antigen‐presenting cells
BMBas: bone marrow‐derived basophils
ECs: epithelial cells
HDM: house dust mite
MLNs: mediastinal lymph nodes
OVA: ovalbumin
OxPAPC: oxidized 1‐palmitoyl‐2‐arachidonyl‐sn‐glycero‐3‐phosphorylcholine
pBECs: primary bronchial epithelial cells
PBS: phosphate buffer saline
TSLP: thymic stromal lymphopoietin
TSLPR: thymic stromal lymphopoietin receptor
EF‐OVA: endotoxin‐free OVA

## Supporting information

Peer review correspondence

**Figure S1**. Contour plots showing the gating scheme for identification of CD4+IL‐4+ T cells from the mediastinal lympho nodes of mice. The right top plots show the CD4+IL‐4+ T cells that were analyzed as Th2 cells.
**Figure S2**. Contour plots showing the gating scheme for identification of eosinophils from the BALF of WT mice and TLR2‐/‐ mice. The final contour plots on the left top show the CD11c‐Siglec F+ cells that were analyzed as indicated by the black rectangular gate.
**Figure S3**. Contour plots showing the gating scheme for identification of basophils from the blood of WT mice and TLR2‐/‐ mice. The final contour plots on the left top show the c‐kit‐FceRI+CD49b+CD200R+cells that were analyzed as indicated by the black rectangular gate.
