# Supplementary material for: Airway epithelial TSLP production of TLR2 drives type 2 immunity in allergic airway inflammation
Source: Eur J Immunol. 2018 Oct 12;48(11):1838–50. doi: 10.1002/eji.201847663 (PMC6282509; doi:10.1002/eji.201847663)
Supplement: Supplementary file 2 — Figure S1. Contour plots showing the gating scheme for identification of CD4+IL‐4+ T cells from the mediastinal lympho nodes of mice. The right top plots show the CD4+IL‐4+ T cells that were analyzed as Th2 cells. Figure S2. Contour plots showing the gating scheme for identification of eosinophils from the BALF of WT mice and TLR2‐/‐ mice. The final contour plots on the left top show the CD11c‐Siglec F+ cells that were analyzed as indicated by the black rectangular gate. Figure S3. Contour plots showing the gating scheme for identification of basophils from the blood of WT mice and TLR2‐/‐ mice. The final contour plots on the left top show the c‐kit‐FceRI+CD49b+CD200R+cells that were analyzed as indicated by the black rectangular gate. [file EJI-48-1838-s002.pdf]

Supplemental Figure 1

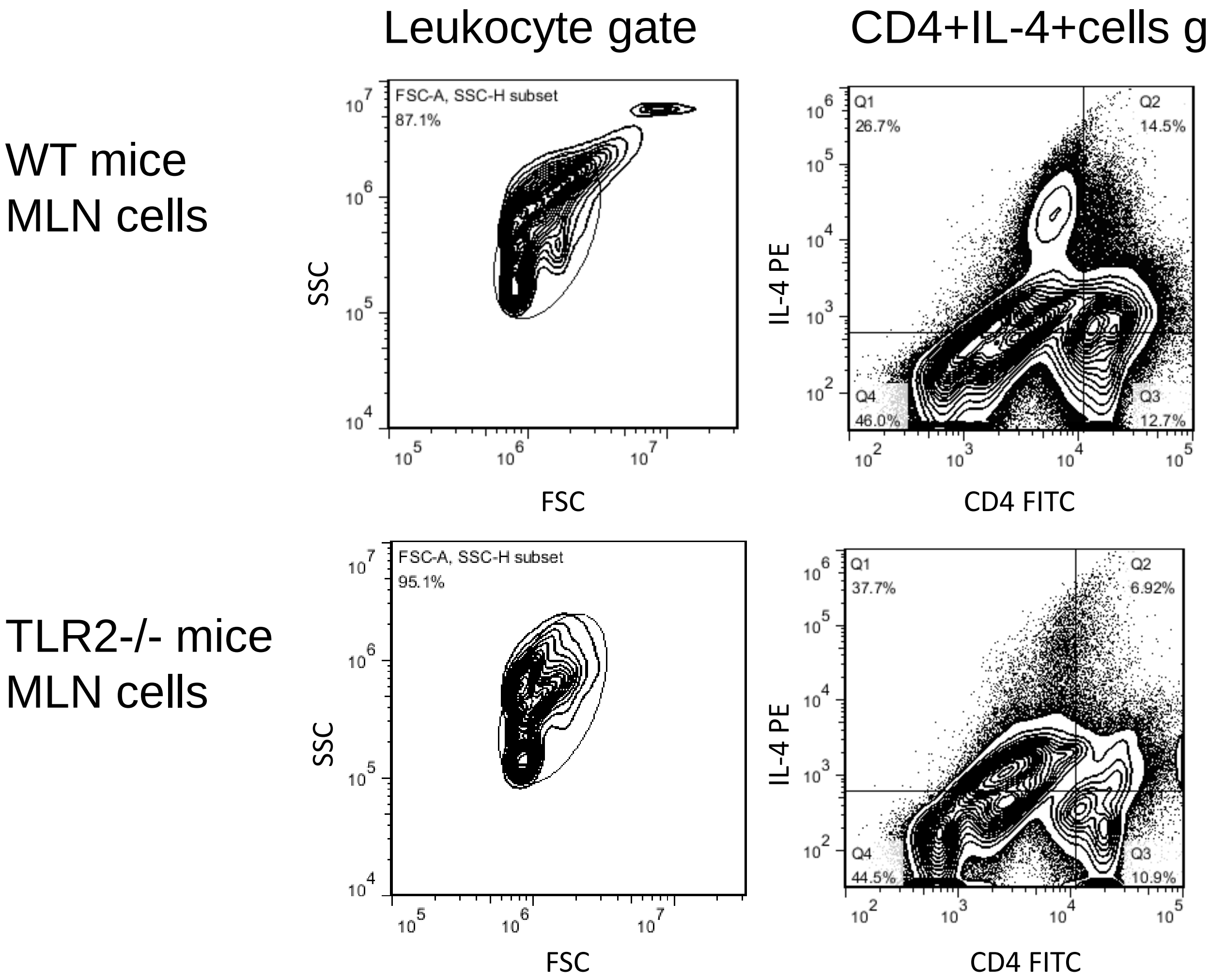

Supplemental Figure 1. Contour plots showing the gating scheme for identification of CD4+IL-4+ T cells from the mediastinal lympho nodes of mice. The right top plots show the CD4+IL-4+ T cells that were analyzed as Th2 cells.

Supplemental Figure 2

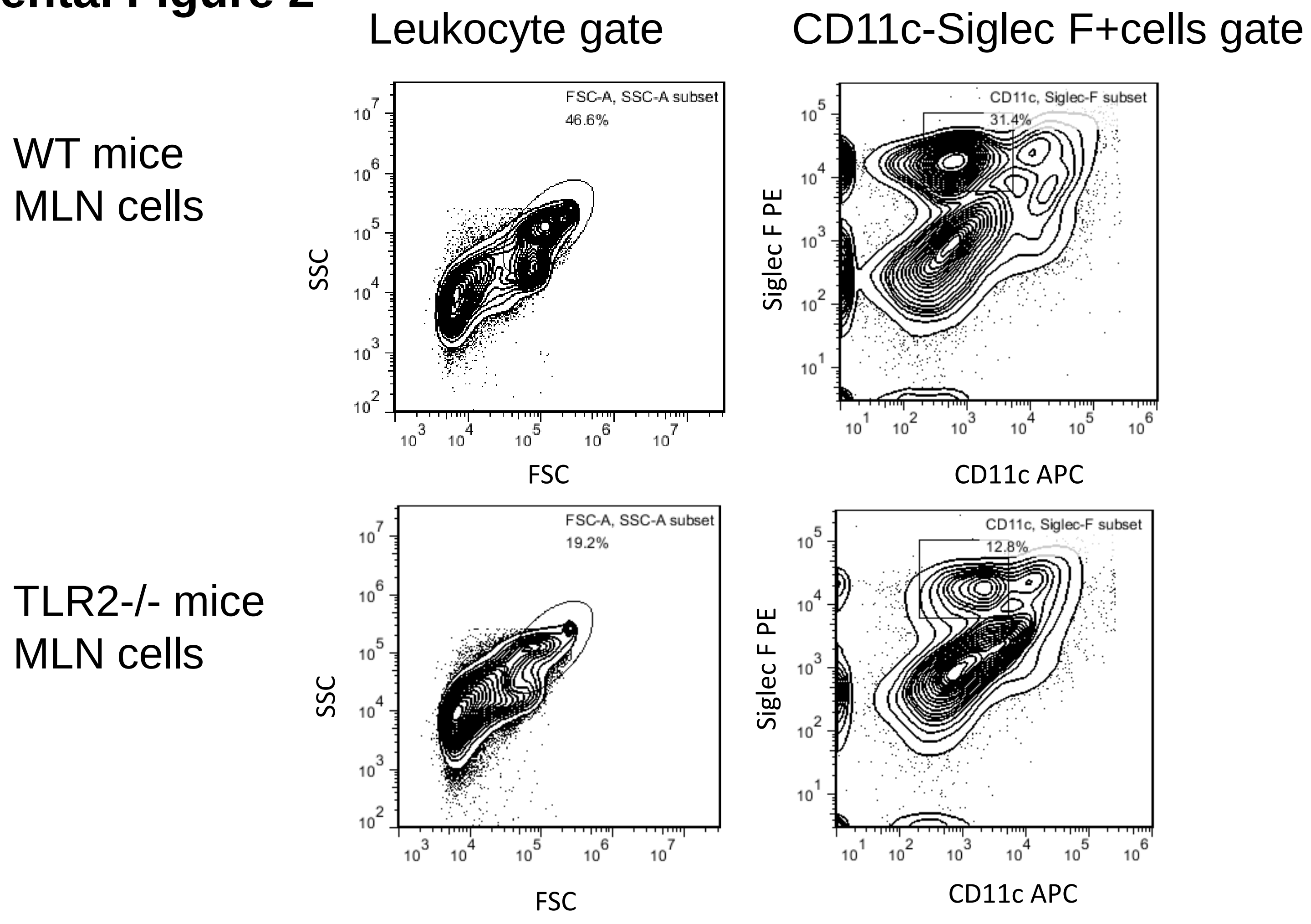

Supplemental Figure 2. Contour plots showing the gating scheme for identification of eosinophils from the BALF of WT mice and TLR2-/- mice. The final contour plots on the left top show the CD11c-Siglec F+cells that were analyzed as indicated by the black rectangular gate.

Supplemental Figure 3

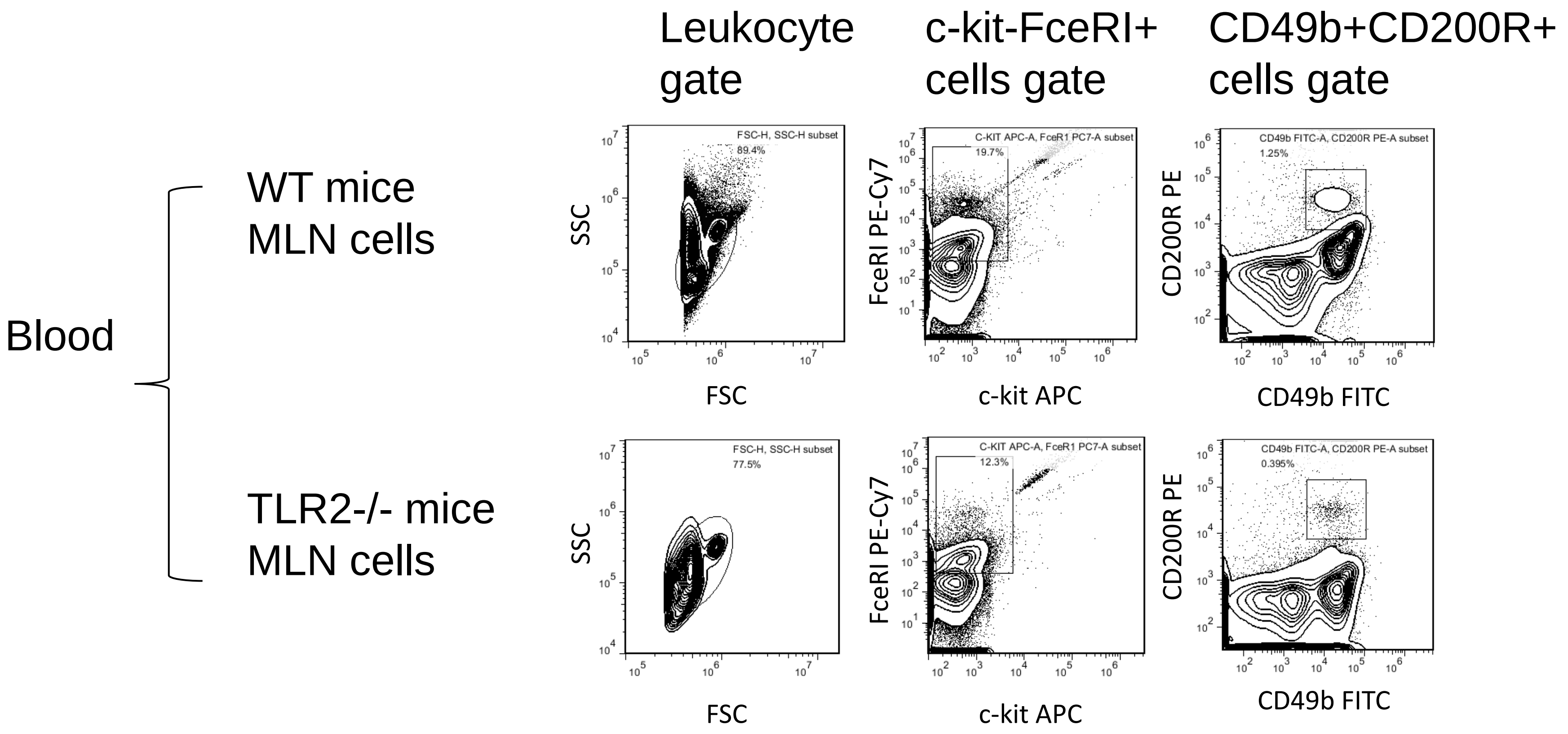

Supplemental Figure 3. Contour plots showing the gating scheme for identification of basophils from the blood of WT mice and TLR2-/- mice. The final contour plots on the left top show the c-kit-FceRI+CD49b+CD200R+cells that were analyzed as indicated by the black rectangular gate.
