# Supplementary material for: Airway epithelial TSLP production of TLR2 drives type 2 immunity in allergic airway inflammation
Source: Eur J Immunol. 2018 Oct 12;48(11):1838–50. doi: 10.1002/eji.201847663 (PMC6282509; doi:10.1002/eji.201847663)
Supplement: Supplementary file 1 — Peer review correspondence [file EJI-48-1838-s001.pdf]

European Journal of Immunology

Airway epithelial TSLP production of TLR2 drives type 2 immunity in allergic airway inflammation

Lv, Jiajia ; Yu, Qianying ; Lv, Jie ; Di, Caixia ; Lin, Xiaoliang ; Su, Wen ; Wu, Min ; Xia, Zhenwei

Correspondence: Zhenwei, Xia, MD, Ph.D. Department of Pediatrics, Ruijin Hospital, Shanghai Jiao Tong University, School of Medicine, Shanghai 200025, China

Review Timeline:	Submission date:	25-Apr-2018
	First Editorial decision:	04-May-2018
	Final Revision received:	29-Jun-2018
	Accepted:	03-Sep-2018

Handling Executive Committee member: Prof. Kenneth Murphy

This manuscript was submitted to EJI with the reviewers' reports and editorial correspondence arising from the earlier peer review process at another journal. We do not have the permission from the reviewers to publish their reports.
